# BEYOND BURDEN: LINKING CAREGIVING EXPERIENCES WITH SELF-PERCEPTIONS OF AGING AND HEALTH BEHAVIORS

**DOI:** 10.1093/geroni/igae098.0862

**Published:** 2024-12-31

**Authors:** Emily Mroz, Shelbie Turner, Becca Levy

**Affiliations:** Chair; Co-Chair; Discussant

## Abstract

Family caregivers to older adults with chronic, progressive illnesses are intimate witnesses to complex diagnoses, symptoms, and medical decisions and the pathways for aging well amidst these challenges. Yet, little research examines whether or how navigating caregiving experiences influence caregivers’ beliefs about their own health, aging, and end-of-life in ways that inform their behaviors (e.g., preparation for their own age-related changes). Aligned with GSA 2024’s theme, this symposium examines caregiving experiences as potential “fortitude factors” that can guide positive aging attitudes and health behaviors. First, Rita Hu will introduce profiles of caregiving trajectories across the adult life course and describe associations between these profiles and caregivers’ self-perceptions of aging. Rachel Bloom will then present a qualitative study of former dementia-caregivers’ observations about the advantages and limitations of medical advocacy and end-of-life care services. She will describe how these observations can be steered by caregivers’ personal narratives from their lived caregiving experiences. Yifan Lou will then build on this by describing quantitative associations between dementia- and non-dementia-caregiving experiences and caregivers’ engagement in advance care planning, describing differences in ACP behaviors across racial and ethnic subgroups. In addition, a fourth presentation will spotlight Roberta Cruz, a family caregiver and member of the National Alzheimer’s Project ACT Advisory Council and IMPACT Collaboratory Lived Experience panel, who will share her perspectives on this emerging topic. Our discussant, Becca Levy, will integrate our presentations with the broader landscapes of research on self-perceptions of aging and caregiver wellbeing, concluding with recommendations for future research.

